# Iron Metabolism in the Colorectal Tumor Microenvironment: Current Evidence and Clinical Implications

**DOI:** 10.3390/diagnostics16132081

**Published:** 2026-07-02

**Authors:** Anamaria-Vlăduța Tomoiagă, Angela Cozma, Cezara-Andreea Gerdanovics, Alexandru Gerdanovics, Mircea-Vasile Milaciu, Nicoleta-Valentina Leach, Vasile Negrean, Șoimița-Mihaela Suciu, Simona Valeria Clichici, Olga Hilda Orășan

**Affiliations:** 1 Department of Internal Medicine, 4th Medical Discipline, “Iuliu Hațieganu” University of Medicine and Pharmacy, Republicii Street, No. 18, 400015 Cluj-Napoca, Romania; vladutat@yahoo.com (A.-V.T.); andreea.ceza.irimie@elearn.umfcluj.ro (C.-A.G.); vasile.milaciu@umfcluj.ro (M.-V.M.); vasile.negrean@umfcluj.com (V.N.); hilda.orasan@umfcluj.ro (O.H.O.); 2 Neurology Department, Clinical Rehabilitation Hospital, Viilor Street, No. 46-50, 400066 Cluj-Napoca, Romania; alexandru.gerdanovics@elearn.umfcluj.ro; 3 Neuroscience Department, “Iuliu Hațieganu” University of Medicine and Pharmacy, 400012 Cluj-Napoca, Romania; 4 Department 2, Faculty of Nursing and Health Sciences, “Iuliu Hațieganu” University of Medicine and Pharmacy, Republicii Street, No. 18, 400015 Cluj-Napoca, Romania; nicoleta_leach@yahoo.com; 5 Department of Physiology, “Iuliu Hațieganu” University of Medicine and Pharmacy, 1-3 Clinicilor Street, 400006 Cluj-Napoca, Romania; ssuciu@yahoo.com (Ș.-M.S.); sclichici@umfcluj.ro (S.V.C.)

**Keywords:** colorectal cancer, iron metabolism, iron dysregulation, functional iron deficiency, hepcidin–ferroportin axis, transferrin saturation, tumor microenvironment, oxidative stress, iron-related biomarkers

## Abstract

Iron is essential for normal cellular function, but its dysregulation is increasingly recognized as a key factor in colorectal tumorigenesis. This review provides an integrated overview of iron-related biomarkers across the full spectrum of colorectal neoplasia, from preneoplastic lesions to advanced colorectal cancer (CRC). Evidence suggests that alterations in iron metabolism begin early, at the level of colorectal adenomas, where increased iron uptake and impaired export contribute to local iron accumulation and oxidative stress. As lesions progress to carcinoma, this imbalance becomes more pronounced, leading to expansion of the intracellular labile iron pool and supporting tumor growth, metabolic adaptation, and genomic instability. At the systemic level, patients often exhibit reduced circulating iron despite preserved or elevated ferritin levels, reflecting inflammation-driven functional iron deficiency. This pattern is largely mediated by dysregulation of the hepcidin–ferroportin axis. In this context, transferrin saturation and soluble transferrin receptor may provide a more accurate assessment of iron availability than ferritin alone. At the tissue level, increased expression of iron import proteins and impaired iron export promote intracellular iron retention. Excess iron further contributes to reactive oxygen species generation, leading to damage of DNA, lipids, and proteins. Clinically, iron-related biomarkers show variable diagnostic performance but may offer prognostic value. Integrating systemic and tissue biomarkers could improve risk stratification and support personalized approaches across the CRC continuum.

## 1. Introduction

Iron is an essential trace element required for many fundamental biological processes, including oxygen transport, DNA synthesis, mitochondrial function, and immune responses [1]. Its ability to switch between ferrous (Fe^2+^) and ferric (Fe^3+^) forms makes it indispensable for cellular activity. However, these same properties also allow iron to generate reactive oxygen species when present in excess, leading to damage of DNA, lipids, and proteins. For this reason, iron levels are tightly regulated, and disruption of iron balance can contribute to disease development, including cancer [2].

In recent years, growing attention has been directed toward the role of iron metabolism in colorectal tumorigenesis. Rather than reflecting a simple increase or decrease in iron levels, colorectal neoplasia is characterized by a progressive reorganization of iron handling at both systemic and cellular levels. Early changes can already be detected in preneoplastic lesions, where alterations in iron uptake and storage contribute to a microenvironment that favors oxidative stress and cellular proliferation [3].

At the cellular level, colorectal tumor cells adapt their iron metabolism to support growth. They increase iron uptake and reduce iron export, leading to accumulation of iron inside the cell. This intracellular iron supports cell proliferation, metabolic activity, and survival, but also promotes oxidative stress and genetic instability [4]. Importantly, these changes are not limited to advanced cancer. Alterations in iron handling can already be detected in early lesions such as adenomas, suggesting that iron may play a role from the initial stages of tumor development [5].

**Figure 1:**
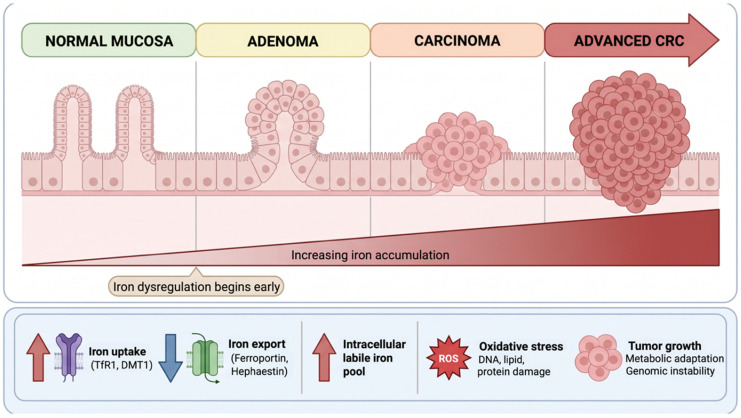
Progressive changes in iron metabolism across the colorectal neoplasia sequence. This figure illustrates the evolution of iron metabolism from normal mucosa to adenoma, carcinoma, and advanced CRC. Abbreviations: TfR1, transferrin receptor 1; DMT1, divalent metal transporter 1; ROS, reactive oxygen species. Created in BioRender. Vladuta, T. (2026) https://BioRender.com/4icbc4w (accessed on 26 April 2026).

Iron metabolism is also closely linked to the tumor microenvironment (TME). Interactions between tumor cells, immune cells, and local factors influence how iron is distributed and used, affecting both tumor progression and immune responses. In this context, iron-dependent processes such as ferroptosis have attracted interest as potential therapeutic targets [6]. As illustrated in Figure 1, these alterations intensify progressively along the neoplastic sequence, with increasing iron uptake and reduced export driving expansion of the labile iron pool and consequent ROS-mediated damage to DNA, lipids, and proteins from the adenoma stage through advanced disease.

From a clinical perspective, iron-related biomarkers may provide useful information for diagnosis and prognosis in CRC. However, their interpretation is challenging because they are influenced by inflammation and do not always reflect iron levels within the tumor [7]. A better understanding of how iron metabolism changes across different stages of disease may improve their clinical value.

This review aims to provide an overview of iron metabolism in CRC, from early lesions to advanced disease, with a focus on its role in tumor biology and its potential as a source of clinically relevant biomarkers.

## 2. Iron Metabolism and Iron-Related Biomarkers in Colorectal Cancer

### 2.1. Iron Handling Within the Tumor Microenvironment

Iron homeostasis within the colorectal TME is orchestrated through dynamic interactions between malignant epithelial cells, immune cells, stromal cells, and inflammatory mediators rather than by tumor cells alone. These interactions continuously regulate local iron availability, linking iron metabolism with chronic inflammation, immune modulation, and tumor progression [8,9].

A central mechanism governing iron trafficking within the TME is the hepcidin–ferroportin axis. Beyond its systemic role in maintaining iron homeostasis, locally produced hepcidin promotes ferroportin internalization and degradation, thereby reducing cellular iron export and favoring iron sequestration within the TME [10,11,12]. In CRC, inflammatory cytokines, particularly IL-6, activate STAT3-dependent hepcidin expression, establishing a positive feedback loop that reinforces local iron retention while contributing to systemic functional iron deficiency (FID) [3,10,13].

Among the non-malignant cellular components of the TME, tumor-associated macrophages (TAMs) are major regulators of iron distribution. Their iron-handling phenotype is highly plastic and adapts to the surrounding inflammatory milieu. While classically activated (M1-like) macrophages preferentially retain intracellular iron as part of their antimicrobial and antitumor functions, alternatively activated (M2-like) macrophages promote iron recycling and redistribution to neighboring tumor cells through multiple iron-export mechanisms, thereby supporting tumor metabolism, angiogenesis, and immune evasion. Consequently, TAMs function not only as immune regulators but also as key mediators of iron exchange between stromal and malignant compartments [14].

**Figure 2:**
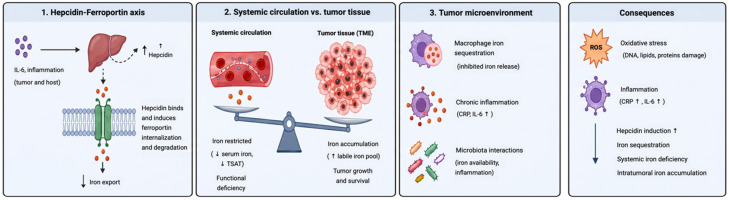
Iron handling within the colorectal TME. (**Panel 1**) shows IL-6/hepcidin-mediated systemic iron restriction. (**Panel 2**) illustrates iron redistribution from the circulation to the tumor. (**Panel 3**) depicts iron sequestration within the TME. The final sectionsummarizes the downstream consequences of iron dysregulation, including LIP expansion, ROS generation, and tumor progression. Abbreviations: IL-6, interleukin-6; CRP, C-reactive protein; Created in BioRender. Vladuta, T. (2026) https://BioRender.com/bvm5rm7 (accessed on 26 April 2026).

Collectively, these findings demonstrate that iron handling within the TME is an active multicellular process integrating inflammatory signaling, immune cell function, and stromal–tumor interactions. The reciprocal relationship between cytokine-driven iron sequestration, macrophage polarization, and local iron redistribution contributes to the characteristic coexistence of systemic iron deficiency and intratumoral iron accumulation in CRC. Therefore, the TME should be regarded not only as a source of iron-related biomarkers but also as a promising therapeutic target for modulating tumor progression and treatment response [3,10,12,13,14]. These interconnected mechanisms are summarized in Figure 2, which illustrates how inflammation-driven iron redistribution links systemic iron restriction with intratumoral iron accumulation, macrophage iron sequestration, and oxidative stress within the colorectal TME.

### 2.2. Intracellular Iron Homeostasis and Ferritinophagy

Maintenance of intracellular iron homeostasis extends beyond iron uptake and export. Once internalized, iron availability is further regulated by ferritin turnover, labile iron pool dynamics, mitochondrial iron metabolism, and ferroptosis-related pathways, which collectively determine the balance between metabolic adaptation and oxidative stress [9,15].

A central mechanism controlling intracellular iron mobilization is ferritinophagy, a selective form of autophagy mediated by nuclear receptor coactivator 4 (NCOA4). Acting as a cargo receptor, NCOA4 delivers ferritin to lysosomes for degradation, allowing the release of ferritin-bound iron into the cytosol and increasing the bioavailable iron pool [15,16]. Through this mechanism, ferritinophagy rapidly adjusts intracellular iron availability according to cellular metabolic demands. However, excessive iron release may also promote ROS formation through Fenton chemistry, increasing lipid peroxidation and enhancing ferroptosis susceptibility [15,16,17].

The labile iron pool (LIP) represents the metabolically active fraction of intracellular iron that fuels essential cellular processes, including DNA synthesis, mitochondrial respiration, and iron-containing enzyme activity. Because this iron fraction is highly redox-active, its expansion must be tightly controlled to prevent excessive oxidative damage [9,15]. Accordingly, ferritin storage, ferritinophagy, and iron transport proteins cooperate to maintain intracellular iron balance while limiting oxidative injury [9,15,16].

Mitochondria play an equally important role in intracellular iron regulation by supporting heme synthesis, iron–sulfur cluster assembly, and oxidative phosphorylation [9,18]. Recent studies further indicate that CRC cells exploit mitochondrial adaptive mechanisms, including the heme–succinate dehydrogenase–coenzyme Q (SDH–CoQ) axis, to buffer iron-induced oxidative stress and preserve mitochondrial function despite persistent intracellular iron accumulation [18].

Emerging evidence suggests that intracellular iron homeostasis may also influence CRC stem cell biology. Although direct evidence in CRC stem cells remains limited, studies in other tumor types have demonstrated that altered ferritinophagy and iron-storage reprogramming contribute to stem cell-like phenotypes, metabolic plasticity, and therapeutic resistance [19,20]. These observations suggest that similar mechanisms may operate in CRC, where dysregulated iron metabolism could support tumor progression and resistance to therapy [19]. Conversely, activation of the NCOA4–ferritinophagy pathway increases intracellular iron availability and may sensitize tumor cells to ferroptosis, suggesting that modulation of ferritinophagy could represent a promising therapeutic strategy. Although these findings remain largely preclinical, targeting intracellular iron homeostasis offers an attractive avenue for future biomarker development and precision therapies in CRC [15,21].

### 2.3. Systemic Biomarkers

Iron deficiency is one of the most common hematologic abnormalities in CRC, affecting approximately 50–64% of patients at diagnosis and occurring more frequently in right-sided tumors. Serum iron levels decline progressively with advancing disease stage, reflecting chronic occult bleeding and inflammation-associated iron restriction. Advanced CRC is consistently associated with significantly lower circulating iron compared with early-stage disease and healthy controls [7].

Interpretation of ferritin in CRC is complex because ferritin functions both as an indicator of iron stores and as an acute-phase reactant. Cancer-related inflammation may elevate ferritin despite true iron deficiency, potentially masking iron depletion. Nevertheless, many CRC cohorts demonstrate lower mean ferritin levels compared with controls, particularly in advanced disease. For this reason, combined iron indices are recommended [22]. However, the diagnostic cutoff values used for ferritin vary considerably across studies and clinical guidelines, ranging from <15 to <100 ng/mL for iron deficiency. Conversely, elevated ferritin has also been associated with poor prognosis in advanced CRC. This variability limits direct comparisons between studies and prevents the establishment of universally accepted CRC-specific diagnostic and prognostic thresholds [23,24]. TSAT is less affected by inflammation and is considered a more reliable marker of iron-restricted erythropoiesis in oncology patients. Current guidelines generally define absolute iron deficiency as ferritin <30 ng/mL together with TSAT <20%, whereas FID, common in CRC, is characterized by normal or moderately elevated ferritin with reduced TSAT [22]. Nevertheless, these thresholds were established primarily for broader cancer populations and differ across international guidelines, while CRC-specific diagnostic criteria remain unavailable [25].

Clinically, iron deficiency in CRC is associated with adverse tumor characteristics, including larger tumor size, more advanced T and N stages, poorer differentiation, and increased lymphovascular and perineural invasion. In the neoadjuvant setting, iron-deficient patients tend to exhibit lower tumor regression grades, and postoperatively they often experience higher inflammatory markers, delayed bowel recovery, and longer hospitalization [7].

Hepcidin, the master regulator of systemic iron homeostasis, plays a central role in CRC-associated iron dysregulation. Although circulating hepcidin levels in CRC may fall within the normal laboratory range, they are frequently inappropriately elevated relative to the degree of systemic iron restriction, reflecting the inflammatory tumor milieu. Importantly, hepcidin expression is detectable within CRC tumor tissue in roughly one-third of cases and correlates positively with tumor stage and ferroportin suppression [10].

In metastatic CRC, elevated baseline serum hepcidin (>40 ng/mL) has been identified as an independent predictor of poorer overall survival, supporting its potential prognostic value. Soluble transferrin receptor (sTfR) levels are typically increased in CRC patients, reflecting heightened cellular iron demand and FID. Because sTfR is minimally influenced by inflammation, the sTfR/ferritin ratio may help distinguish true iron deficiency from anemia of chronic disease in the cancer setting [26]. Despite their encouraging evidence supporting their diagnostic and prognostic potential, both hepcidin and sTfR currently lack standardized analytical methods and validated diagnostic or prognostic cutoff values. Differences among ELISA platforms, assay methodologies, and the influence of inflammation, hypoxia, and tumor stage complicate direct comparisons across studies and limit their routine clinical implementation [10,12,26]. Consequently, prospective multicenter validation and harmonization of analytical protocols are required before these biomarkers can be incorporated into precision medicine strategies for CRC [27].

### 2.4. Tissue Biomarkers

At the tissue level, CRC demonstrates consistent dysregulation of iron transport proteins. TfR1 is frequently overexpressed in tumor tissue and supports increased iron uptake required for tumor proliferation. Expression tends to be highest in early-stage, well-differentiated tumors, suggesting a role in tumor establishment, although altered patterns may emerge in advanced disease [28].

Ferroportin, the only known cellular iron exporter, shows functional impairment in CRC. Despite detectable expression, ferroportin is often mislocalized intracellularly rather than positioned on the cell membrane, effectively limiting iron efflux and promoting intracellular iron retention. Reduced expression of its partner protein hephaestin further contributes to defective iron export. Loss of effective ferroportin activity has been associated with more advanced tumor stage [7].

STEAP4, a ferrireductase involved in mitochondrial iron handling, is upregulated in CRC and in inflammation-associated colorectal tumorigenesis. Increased STEAP4 expression correlates with poorer prognosis and greater tumor burden in experimental and clinical studies, highlighting its potential role as both biomarker and therapeutic target [29].

Histochemical analysis using Perls’ Prussian blue staining demonstrates increased intratumoral iron deposition in approximately 30% of CRC cases. Iron accumulation is more commonly observed in right-sided tumors and correlates with higher T stage. Notably, systemic iron deficiency may coexist with local tumor iron loading, suggesting preferential sequestration of iron within the TME [30].

### 2.5. Oxidative Stress and Inflammatory Markers

Markers of oxidative protein damage are consistently elevated in CRC. Protein carbonyl (PCO) levels are significantly higher in both plasma and tumor tissue of CRC patients compared with controls and show positive associations with disease presence and stage. Mitochondrial PCO appear particularly sensitive indicators of tumor-associated oxidative stress. Importantly, systemic PCO concentrations decline following tumor resection, supporting the tumor as a major contributor to systemic oxidative burden [31].

Inflammatory markers are tightly linked to altered iron homeostasis in CRC. Serum CRP and IL-6 are consistently elevated and increase with advancing stage. Both markers independently predict poorer outcomes and reflect the systemic inflammatory response accompanying CRC [32].

**Figure 3:**
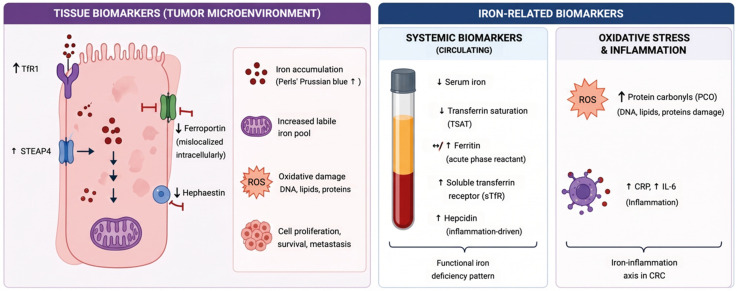
Iron-related biomarkers and associated mechanisms in CRC. The schematic summarizes the principal systemic, tissue, oxidative stress, and inflammatory biomarkers associated with disrupted iron metabolism in CRC. Systemic iron restriction coexists with intratumoral iron accumulation, supporting tumor progression and providing complementary diagnostic and prognostic information. Abbreviations: STEAP4, six-transmembrane epithelial antigen of prostate 4. Created in BioRender. Vladuta, T. (2026) https://BioRender.com/e5d3m7z (accessed on 26 April 2026).

IL-6-driven hepcidin induction contributes to iron sequestration, creating the characteristic pattern in which systemic iron deficiency coexists with intratumoral iron accumulation. Clinically, CRC patients with iron deficiency frequently exhibit higher CRP levels, lower albumin, more aggressive tumor biology, and impaired postoperative recovery. Together, the iron–inflammation axis represents an important biomarker network with diagnostic, prognostic, and potential therapeutic relevance in CRC, as summarized in Figure 3 [3].

### 2.6. Translational Value of Iron Biomarkers

Iron biomarkers show heterogeneous diagnostic performance in colorectal neoplasia, with stronger utility for advanced disease detection than for early screening. Among circulating indices, serum ferritin and TSAT demonstrate the most consistent associations with colorectal adenomas and cancer, although interpretation is limited by inflammatory confounding and biological variability [22].

In colorectal adenomas, TSAT appears to be the most informative marker. Elevated TSAT (>40%) is associated with increased risk of both overall and advanced adenomas, suggesting that systemic iron availability may reflect pathways linking iron exposure to early neoplastic transformation [22]. Some studies report lower serum iron and ferritin in patients with large adenomas (≥1 cm), possibly due to occult bleeding; however, meta-analyses show inconsistent relationships between conventional iron indices and adenoma risk, limiting their value for population screening [33].

In CRC, iron biomarkers have greater diagnostic relevance, particularly in advanced disease. Patients with advanced CRC frequently exhibit reduced serum iron and ferritin compared with healthy controls, and combined low iron–low ferritin patterns are common. Multimarker approaches improve performance: when ferritin is combined with tumor-associated proteins such as CEA and inflammatory markers, diagnostic accuracy increases substantially compared with single-marker approaches [34].

Iron status also carries important prognostic information in CRC, characterized by a U-shaped relationship in which both deficiency and excess are associated with adverse outcomes. Low preoperative iron has been independently associated with shorter overall survival and inferior response to neoadjuvant therapy, as well as delayed postoperative recovery and heightened systemic inflammation [34].

Conversely, iron overload markers also predict worse prognosis. Elevated TSAT (>40%) and high ferritin levels have been linked to reduced survival in stage II–III and metastatic CRC. High iron status has additionally been associated with increased intratumoral Fusobacterium nucleatum, suggesting interactions between iron availability and the tumor microbiome [35].

Emerging evidence supports a predictive role for iron biomarkers in treatment response. Iron deficiency is associated with poorer response to chemoradiotherapy and may impair antitumor immunity, potentially influencing immunotherapy outcomes. In the perioperative setting, iron deficiency predicts slower recovery and worse nutritional–inflammatory profiles, supporting its inclusion in patient blood management strategies [36].

Despite growing clinical interest, implementation remains limited by major barriers. Assay standardization, particularly for sTfR, and wide variability in diagnostic thresholds complicate interpretation. Ferritin is strongly influenced by inflammation, while serum iron and TSAT are affected by fasting status and recent supplementation. Real-world practice gaps are substantial, with under-testing and under-treatment of iron deficiency frequently reported in CRC populations. Greater harmonization of assays, clearer guideline consensus, and prospective biomarker-driven studies are needed to define the optimal role of iron indices across the CRC continuum [3,7,37].

## 3. Clinical Implications of Iron Dysregulation

**Figure 4:**
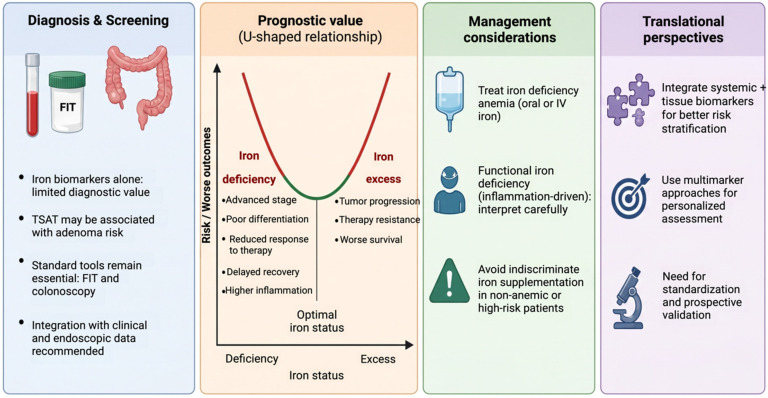
Clinical implications of iron dysregulation in CRC. The schematic summarizes the impact of iron dysregulation on diagnosis, prognosis, iron deficiency classification, therapeutic decision-making, and personalized management in CRC. Integration of systemic and tissue biomarkers may improve risk stratification and support biomarker-guided clinical management. Abbreviations: FIT, fecal immunochemical test. Created in BioRender. Vladuta, T. (2026) https://BioRender.com/2dqqioa (accessed on 26 April 2026).

Iron dysregulation in CRC extends beyond a mere epiphenomenon of systemic inflammation or nutritional imbalance and reflects complex interactions between tumor biology and host iron homeostasis. Clinical observations have consistently highlighted the paradoxical coexistence of systemic iron deficiency and intratumoral iron accumulation, underscoring the need to interpret iron-related biomarkers within a broader biological and clinical context [3]. Alterations in circulating markers such as ferritin, TSAT, and hepcidin may therefore provide incomplete or even misleading information when considered in isolation, while tissue-level changes in iron transport and storage pathways appear to more closely mirror tumor aggressiveness and disease progression [38]. Understanding the clinical implications of iron dysregulation is essential for accurate risk stratification, prognostic assessment, and informed decision-making in patients with CRC, as summarized in Figure 4.

### 3.1. Prognostic Value of Iron-Related Biomarkers

Accumulating evidence suggests that alterations in iron-related biomarkers are associated with tumor aggressiveness and adverse clinical outcomes in CRC [3,38]. Elevated serum ferritin levels, traditionally regarded as markers of iron stores or systemic inflammation, have been linked to advanced tumor stage, increased tumor burden, and poorer survival, although their interpretation remains challenging due to confounding inflammatory signals [38]. In parallel, dysregulation of hepcidin expression has emerged as a key determinant of iron sequestration, contributing to FID at the systemic level while favoring iron retention within tumor tissue [39].

Overexpression of TfR1 and downregulation of ferroportin have been consistently associated with enhanced intracellular iron availability, increased proliferative capacity, and metastatic potential. These alterations appear to reflect a tumor-adaptive phenotype characterized by iron trapping and resistance to iron export, conferring a growth advantage under conditions of metabolic and oxidative stress. Importantly, tissue-based iron markers have demonstrated stronger associations with disease progression and prognosis than circulating parameters, highlighting the limitations of relying solely on systemic biomarkers [3].

Taken together, iron-related biomarkers provide complementary prognostic information when interpreted in an integrated manner, combining systemic indices with tissue-level alterations. Such an approach may improve risk stratification by distinguishing patients in whom altered iron homeostasis represents a marker of aggressive tumor biology from those in whom it primarily reflects inflammatory or nutritional factors.

### 3.2. Iron Metabolism and Tumor Stage, Grade, and Metastasis

Dysregulation of iron metabolism has been increasingly associated with CRC progression and correlated with advanced tumor stage, higher histological grade, and metastatic dissemination [3,40]. Several studies have reported that alterations in iron transport and storage pathways may intensify along the adenoma–carcinoma sequence [3,39,40], suggesting a progressive reprogramming of iron handling as tumors acquire more aggressive phenotypes. In particular, increased expression of TfR1 and reduced ferroportin levels have been more frequently observed in advanced-stage tumors, reflecting enhanced iron uptake and impaired iron export [3].

Intratumoral iron accumulation has been linked to adverse disease features and poor prognosis in CRC. For example, iron deposition in tumor tissues has been associated with worse overall outcomes in CRC patients, particularly in specific molecular contexts, such as the presence of Fusobacterium nucleatum [41]. This iron-enriched microenvironment may support metastatic potential by sustaining proliferative signaling, promoting oxidative stress-induced genomic instability, and facilitating metabolic flexibility under hypoxic conditions. Moreover, iron-driven activation of redox-sensitive pathways may contribute to epithelial–mesenchymal transition and increased invasive capacity, linking iron availability to key mechanisms of tumor spread [42,43].

Systemic iron-related biomarkers have also shown associations with disease stage, although these relationships appear less consistent and are often confounded by inflammation and tumor burden [44]. Elevated ferritin levels and altered hepcidin expression have been more commonly reported in patients with advanced or metastatic disease, yet their prognostic value remains inferior to tissue-based markers [38]. Collectively, these observations indicate that iron metabolism is dynamically modulated during CRC progression and that iron-related alterations may serve as indicators of tumor aggressiveness rather than mere reflections of systemic iron status.

### 3.3. Iron Deficiency Anemia Versus Functional Iron Deficiency in CRC

Iron deficiency is highly prevalent in CRC, and both absolute iron deficiency (AID) and FID can coexist in the same patient [45,46]. In clinical cohorts of CRC, iron deficiency has been identified in a substantial proportion of patients when a complete iron panel is available, with AID commonly defined by low ferritin together with low TSAT, while FID is characterized by low TSAT despite ferritin values that are normal or increased (reflecting preserved stores but restricted availability) [44,45].

From a pathophysiological perspective, AID in CRC is most often driven by chronic gastrointestinal blood loss and depleted iron stores, whereas FID is mainly mediated by inflammation (e.g., IL-6) with hepcidin upregulation and ferroportin inhibition, leading to iron sequestration in macrophages/enterocytes and reduced circulating iron available for erythropoiesis [3,38,44,47]. This explains the typical “paradox” pattern in FID: low serum iron and TSAT with ferritin that may be >100 μg/L [47].

Clinically, separating AID from FID matters because systemic biomarkers behave differently in inflammation and because mixed phenotypes are frequent in CRC. For example, a large preoperative CRC cohort reported that most iron deficiency cases reflected a combination of AID and FID, and iron deficiency was more likely with more advanced pTNM stage and right-sided tumor location [45]. In another CRC cohort, FID was common and showed an association with lymphatic invasion, supporting the idea that FID may coexist with more aggressive disease features in at least some populations [48].

The frequent coexistence of absolute and FID, CRC together with the inflammatory modulation of systemic iron biomarkers, complicates anemia management and underscores the need for careful evaluation before initiating iron supplementation or other iron-modulating strategies.

### 3.4. Potential Risks and Benefits of Iron Supplementation

Iron supplementation represents a cornerstone of supportive care in CRC patients with iron deficiency anemia, particularly in the perioperative setting, where correction of anemia has been associated with improved functional status and reduced transfusion requirements. Both oral and intravenous iron formulations have demonstrated efficacy in increasing hemoglobin levels in iron-deficient CRC patients, especially when iron deficiency is clearly documented [47,48,49].

Although iron supplementation is generally recommended for patients with confirmed iron deficiency anemia, its use should be individualized according to iron status, inflammatory activity, and the underlying clinical context. The potential effects of iron supplementation on oxidative stress, the TME, non-anemic individuals, and early tumorigenic processes remain areas of active investigation and are discussed in detail in Section 4. Current evidence does not support routine iron supplementation in non-anemic patients with CRC, particularly in the absence of documented iron deficiency [50,51].

## 4. Iron Supplementation and Modulation of the Tumor Microenvironment

Iron supplementation represents a cornerstone in the management of iron deficiency and anemia across a wide range of clinical settings. However, beyond its hematological benefits, increasing evidence, shown in the previous section, suggests that iron availability may exert broader biological effects, particularly in conditions characterized by chronic inflammation or altered cellular iron handling. As a result, iron supplementation, especially when administered in the absence of overt anemia, has raised concerns regarding its potential impact on tumor biology and early carcinogenic processes. This section explores the complex relationship between iron supplementation and the TME, focusing on oxidative stress, effects in non-anemic individuals, potential interactions with preneoplastic lesions, and ongoing clinical controversies. Most of the available evidence linking iron supplementation with tumor progression derives from experimental models or mechanistic studies. At present, prospective clinical studies demonstrating that iron supplementation promotes CRC development or progression in humans are lacking. Therefore, the potential biological effects discussed below should be interpreted as mechanistic hypotheses supported primarily by preclinical evidence rather than established clinical observations.

### 4.1. Oral Iron and Oxidative Stress

Oral iron supplementation, particularly ferrous formulations, has been linked to increased oxidative stress locally within the gastrointestinal tract. A relevant mechanistic pathway is that a meaningful fraction of orally administered iron remains unabsorbed and reaches the colon, where iron can catalyze free-radical generation in luminal contents; in healthy volunteers, ferrous sulfate supplementation increased the free radical-generating capacity of feces, supporting the concept that unabsorbed iron can amplify ROS exposure at the mucosal interface [52]. Broader reviews also summarize experimental evidence that excess luminal iron can harm the intestinal mucosa via oxidative stress and barrier-related effects [36].

These local redox effects are clinically relevant because oxidative stress can translate into epithelial irritation and inflammatory signaling, particularly in settings where mucosal vulnerability already exists (e.g., inflammatory bowel disease). A classic clinical discussion in ulcerative colitis highlights that iron supplementation may worsen disease activity, with ROS generation proposed as a contributing mechanism [53]. More recent hematology-focused reviews similarly emphasize that oral iron commonly causes gastrointestinal side effects and that luminal exposure is a key differentiator from parenteral iron—consistent with a local, exposure-driven biological effect rather than a purely systemic phenomenon [54].

Importantly, the magnitude of oxidative stress associated with oral iron appears to depend on the formulation used. Conventional ferrous salts are characterized by low fractional absorption and substantial luminal iron exposure, whereas newer ferric complexes and sucrosomial or liposomal iron formulations have been designed to improve tolerability and limit free iron availability in the intestinal lumen, potentially reducing redox-related mucosal effects, although comparative data on long-term oxidative and oncologic outcomes remain limited [54].

Whether oral iron reliably increases systemic oxidative stress appears less consistent across populations and designs. For example, in a bariatric surgery cohort, a preliminary study reported that high-dose oral iron for iron deficiency did not adversely impact systemic oxidative stress, underscoring that systemic signals may depend on baseline status, inflammation, dosing, and follow-up duration [55].

At the same time, other clinical investigations have examined oxidative stress/DNA damage-related endpoints before and after oral iron therapy in iron deficiency anemia, supporting continued interest in monitoring redox biology in supplemented patients [56].

Human mechanistic data showing increased luminal ROS generation with oral ferrous iron, alongside experimental and translational evidence linking excess luminal iron to mucosal oxidative stress and barrier perturbation, provide a plausible biological basis for downstream effects on inflammation-related pathways relevant to early neoplastic processes and, conceptually, the TME. However, these findings should not be interpreted as evidence that oral iron supplementation accelerates CRC progression in humans, as prospective clinical data addressing this question remain unavailable [54].

### 4.2. Effects of Iron Supplementation in Non-Anemic Patients

Iron supplementation in non-anemic individuals remains a clinically controversial practice, often initiated to “optimize” biochemical indices (e.g., low or low-normal ferritin) rather than to correct overt anemia. While iron deficiency without anemia has been associated with symptoms such as fatigue in selected groups, randomized trials in non-anemic menstruating women show at most modest, symptom-focused benefits, with limited or no consistent impact on broader patient-reported domains (quality of life, mood) or objective functional outcomes [57,58].

From a physiological perspective, non-anemic individuals generally maintain adequate hemoglobin synthesis and oxygen delivery, raising questions regarding the necessity and risk–benefit balance of exogenous iron administration [57,58,59]. In inflammatory contexts, this dilemma becomes sharper: hepcidin, upregulated by inflammation, reduces intestinal iron absorption and traps iron within macrophages via ferroportin internalization, favoring “iron-restricted erythropoiesis” without necessarily reflecting a true deficit in total body iron stores [59]. In such settings, iron supplementation may increase circulating or storage iron without delivering proportional hematologic benefit, and may increase the probability of redox-active iron species under certain conditions.

Mechanistically, concerns extend beyond erythropoiesis because redox-active iron pools, particularly non-transferrin-bound iron (NTBI) under high TSAT, can catalyze free radical generation and oxidative tissue injury. Although NTBI is classically emphasized in iron overload states, its conceptual relevance here is that excess or poorly regulated iron (including after iron administration in susceptible contexts) may increase the fraction of potentially reactive circulating iron, thereby supporting oxidative stress biology [60].

From a TME perspective, increased iron availability may influence key cellular components beyond the malignant clone itself. Macrophage iron handling is tightly coupled to immune phenotype; experimental evidence indicates that iron loading can shift macrophage polarization markers toward a type-2/M2-like response (e.g., increased Arg1/Ym1 and altered cytokine balance), a pattern broadly consistent with pro-tumoral immune programming [61]. In parallel, tumor-microenvironment-focused reviews describe how cancer cells and stromal/immune cells compete for iron, and how iron trafficking within macrophages (including iron-release phenotypes in tumor-associated macrophages) may support tumor growth, angiogenesis, and immune suppression [62].

Taken together, available clinical trials suggest that short-term iron supplementation may provide symptomatic benefit in selected non-anemic individuals with iron deficiency. Nevertheless, concerns regarding oxidative stress, immune modulation, and potential tumor-promoting effects are supported predominantly by mechanistic and experimental studies, whereas prospective clinical evidence demonstrating adverse oncologic outcomes in patients with CRC remains lacking [57,58,62].

### 4.3. Potential Impact on Preneoplastic Lesions

Emerging evidence from experimental models suggests that iron exposure at the level of the intestinal mucosa may contribute to changes in the microenvironment that promote preneoplastic lesion development. In a recent murine model using Apc^Min/+ mice, dietary iron supplementation was shown to promote colorectal tumorigenesis in the presence of cancer-derived gut microbiota, with distinct microbiota changes linked to increased tumor burden compared to controls. This study highlights the interaction between iron supplementation, initial microbiota composition, and tumor development in genetically susceptible hosts [63].

Mechanistically, iron may influence epithelial proliferation and carcinogenesis through alterations in gut microbial communities. Increased luminal iron availability has been associated with shifts toward pro-inflammatory and potentially genotoxic bacterial taxa, reductions in beneficial taxa, and enhanced mucosal inflammatory signaling in rodent models. These microbial alterations are proposed to create a pro-tumorigenic microenvironment that favors epithelial turnover and oxidative processes implicated in early neoplastic transformation [63].

Further supporting a link between iron and tumor-related biology, a recent dietary intervention study found that excess iron intake in mice was associated with increased tumor burden and changes in the expression of proteins involved in cell cycle regulation, oxidative stress responses, and gastrointestinal disease pathways in intestinal tumors compared with adequate iron diets [64]. These expression changes suggest that excess iron may modify tumor biology at the tissue level.

Human data directly examining iron supplementation and preneoplastic lesions remain limited, but recent work on gut microbiota characteristics in CRC patients highlights the dynamic interplay between iron metabolism, microbiome composition, and disease progression. In a large metagenomic and metabolomic analysis of individuals with CRC, altered microbial communities correlated with iron deficiency states and metabolites associated with inflammation and disease severity, suggesting a potential link between iron–microbiota interactions and tumor progression [65].

Collectively, these findings indicate that excess iron exposure may influence early tumorigenic processes in experimental models, particularly in the presence of altered microbiota or genetic susceptibility. However, current evidence is derived predominantly from preclinical or observational studies and does not demonstrate that iron supplementation promotes the development or progression of preneoplastic colorectal lesions in humans. Well-designed prospective clinical studies are required before such mechanistic observations can be translated into clinical recommendations.

### 4.4. Clinical Controversies and Unmet Needs

The findings discussed in Section 4.1, Section 4.2 and Section 4.3 highlight several unresolved clinical controversies and knowledge gaps regarding iron supplementation beyond its established role in the treatment of iron-deficiency anemia. As outlined in Section 4.1, oral iron supplementation, particularly conventional ferrous formulations, can increase luminal iron exposure and oxidative stress, raising concerns about tissue-level effects that are not captured by standard hematologic endpoints.

These biological considerations become especially relevant in non-anemic individuals, as discussed in Section 4.2. In such settings, iron supplementation is often initiated based on biochemical indices rather than functional impairment, despite inconsistent evidence of clinical benefit. The potential for increased redox-active iron and immune modulation in the absence of clear hematologic necessity underscores the need for more cautious patient selection and clearer therapeutic thresholds.

Section 4.3 further extends these concerns by illustrating how excess iron exposure at epithelial interfaces may influence microenvironmental factors implicated in preneoplastic lesion development, particularly in the gastrointestinal tract. Although current evidence is largely experimental or observational, the convergence of oxidative stress, inflammatory signaling, and microbiota alterations provides a biologically plausible framework linking iron availability to early tumor-related processes.

Overall, iron supplementation remains the standard of care for treating iron deficiency and iron-deficiency anemia. Nevertheless, its potential effects on the TME, oxidative stress, and early carcinogenic processes are currently supported predominantly by experimental and mechanistic evidence rather than prospective clinical studies. Future clinical trials should incorporate tissue biomarkers, oxidative stress parameters, microbiome analyses, and long-term oncologic outcomes to better define the safety profile of iron supplementation in patients at risk for colorectal neoplasia. Until such evidence becomes available, iron supplementation should continue to follow established clinical indications, with particular caution in non-anemic individuals and populations at increased risk of CRC.

## 5. Risk Stratification and Translational Perspectives

Recent advances in CRC research emphasize the need for refined risk stratification approaches that extend beyond traditional clinicopathological parameters and incorporate biologically relevant molecular features. Growing evidence indicates that alterations in iron metabolism represent a recurrent hallmark of CRC, with dysregulated iron handling contributing to oxidative stress, inflammatory signaling, and tumor cell proliferation. Importantly, contemporary reviews highlight that changes in iron-related pathways may be observed across different stages of colorectal tumorigenesis, supporting the potential translational relevance of iron-associated biomarkers for risk assessment and preventive strategies [3].

### 5.1. Molecular Heterogeneity and Tumor Location

Right-sided and left-sided CRC display fundamentally distinct molecular architectures that may influence iron metabolism. Right-sided CRC is more frequently characterized by BRAF mutations, deficient mismatch repair (dMMR), microsatellite instability (MSI-H), high tumor mutational burden, and enrichment of the CMS1/CMS3 molecular subtypes, whereas left-sided CRC is typically associated with chromosomal instability (CIN), APC and TP53 mutations, microsatellite stability (MSS), and the CMS2 subtype [66,67]. Beyond the traditional right-versus-left classification, Mendes Oliveira et al. demonstrated substantial molecular heterogeneity across all six colonic segments, identifying only 15 genes commonly mutated throughout the colon, including the canonical drivers APC, PIK3CA, and TP53, while numerous additional mutations displayed segment-specific distributions, emphasizing that anatomical location represents an important determinant of CRC biology [68].

These molecular differences may also contribute to location-specific iron handling and oxidative stress responses. Iron deficiency is significantly more common in right-sided tumors [30,69], while CRC cells generally exhibit increased expression of iron import proteins (TfR1, DMT1, and DCYTB) together with reduced iron export through ferroportin and hephaestin, promoting intracellular iron accumulation and iron-dependent proliferation [3,70]. Because DMT1-mediated iron uptake interacts with JAK/STAT3 signaling [71], and epigenetic regulation differs between CIMP-high right-sided tumors and CIN-dominant left-sided tumors [72,73], the underlying molecular landscape may influence iron homeostasis, oxidative stress adaptation, and ferroptosis susceptibility [74]. These observations support considering tumor sidedness and molecular heterogeneity when evaluating iron-related biomarkers and developing iron-targeted therapeutic strategies in CRC [66,75].

### 5.2. Integrating Systemic and Tissue Iron Biomarkers

Effective risk stratification in CRC requires understanding how different iron biomarkers reflect underlying biology, including systemic measurements (e.g., serum iron, ferritin, TSAT versus local tissue iron handling, iron accumulation or regulatory protein expression within tumor tissue). Research in recent years highlights that systemic iron indices alone show inconsistent associations with CRC risk, whereas local alterations in iron metabolism and iron-related molecules within the TME may provide additional and potentially more relevant information for stratifying risk and understanding disease mechanisms [3,38].

Epidemiological evidence indicates that common systemic iron measures, such as serum iron, ferritin, and TSAT, do not consistently correlate with CRC risk in population studies. For example, a large cross-sectional analysis of U.S. adults using NHANES data from 2001–2020 found no statistically significant association between serum iron status markers and the presence of CRC after adjustment for confounders, although non-significant trends were observed suggesting possible gradients for some markers like ferritin and TSAT [76].

In contrast, tumor tissue and cellular iron regulation pathways show clearer biological relevance in CRC pathogenesis. Recent narrative reviews summarizing molecular evidence indicate that CRC cells often exhibit dysregulated expression of key iron-handling proteins, including elevated TfR1 expression and altered ferritin and ferroportin levels, reflecting increased iron demand and disrupted local iron homeostasis in the TME [3]. These tissue-level alterations are mechanistically linked to processes relevant for neoplastic progression, such as oxidative stress, inflammation, and cell proliferation.

Moreover, clinical studies investigating the impact of iron status on CRC outcomes suggest that iron deficiency, which is common in CRC patients and may manifest with complex interactions between systemic iron availability and tumor biology, can influence therapeutic responses and patient prognosis, underscoring that integrative approaches combining systemic and tissue biomarkers could capture biologically distinct aspects of iron dysregulation [7].

Together, these findings underscore that systemic iron biomarkers alone have limited predictive utility for CRC risk, and that incorporating tissue-level biomarkers of iron metabolism may improve stratification models by reflecting local tumor biology more directly. Future work should focus on validating whether composite biomarker panels that integrate systemic and tissue measures enhance risk prediction and prognostic assessment in CRC.

### 5.3. Identifying High-Risk Preneoplastic Lesions

**Figure 5:**
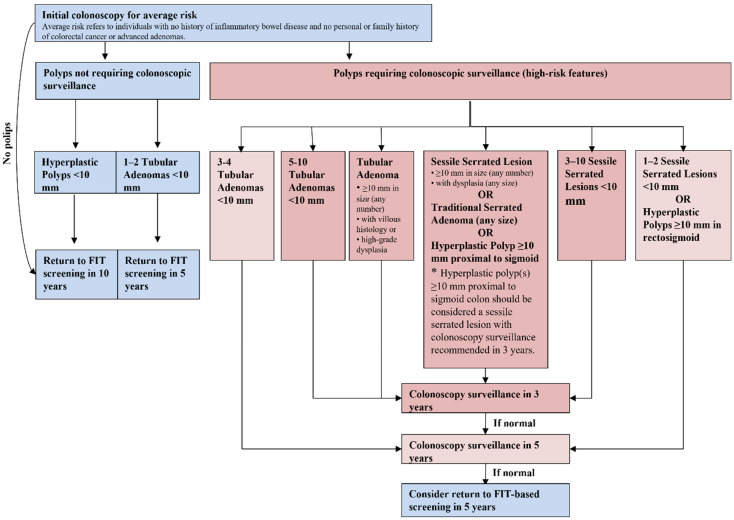
Endoscopic risk stratification of preneoplastic colorectal lesions following initial colonoscopy. The figure summarizes a practical, guideline-based framework for identifying preneoplastic colorectal lesions requiring colonoscopic surveillance, integrating lesion number, size, histological features, and serrated pathway criteria. The schematic highlights high-risk lesion categories that inform surveillance intensity and clinical management decisions. Adapted from Sadowski et al. [78].

Risk stratification at the preneoplastic stage of colorectal carcinogenesis is primarily based on the identification of lesions with a well-established increased likelihood of malignant progression. Current evidence consistently classifies advanced conventional adenomas, defined by a size ≥10 mm, villous or tubulovillous architecture, and/or high-grade dysplasia, as high-risk lesions. [77] In parallel, lesions arising from the serrated pathway, particularly sessile serrated lesions measuring ≥10 mm or those exhibiting dysplasia, are recognized as clinically relevant precursors associated with an increased risk of CRC [77,78]. Figure 5 illustrates a practical, guideline-based framework for endoscopic risk stratification, highlighting the lesion characteristics that identify polyps requiring colonoscopic surveillance. These lesion categories represent the foundation of contemporary post-polypectomy surveillance strategies and are uniformly acknowledged across recent guideline updates and expert reviews.

By translating guideline-defined criteria into a structured endoscopic framework, this approach facilitates reproducible identification of high-risk preneoplastic lesions at the time of colonoscopy. The integration of lesion number, size, serrated pathway features, and histological risk markers supports consistent risk categorization across diverse clinical settings. Such standardized lesion stratification provides a practical basis for individualized surveillance planning and creates a direct link between endoscopic findings and downstream preventive strategies.

### 5.4. Iron Metabolism as a Target for Personalized Prevention Strategies

Recent translational reviews describe altered iron homeostasis as a recurring feature across colorectal carcinogenesis and emphasize its potential relevance for individualized risk assessment rather than as a standalone preventive intervention [79].

From a prevention standpoint, contemporary evidence syntheses highlight that the relationship between iron and CRC risk is complex and not sufficiently consistent to justify iron-targeted chemoprevention [79,80,81]. Reviews note that higher dietary exposure to heme iron (typically from red/processed meat) has been linked to increased CRC risk in multiple lines of evidence, but findings vary by population, exposure assessment, and outcome definitions; recent cohort analyses also illustrate that associations with heme/nitrosyl-heme may be modest or non-significant in some settings [80]. Therefore, the defensible “prevention” implication at present is not active iron modulation, but cautious risk-avoidance principles within broader dietary prevention guidance (e.g., avoiding excessive processed meat intake), rather than prescribing iron restriction.

Importantly, the current literature does not support routine iron supplementation or iron-modulating agents as strategies to prevent colorectal neoplasia in unselected individuals. Recent reviews instead emphasize that iron interventions are primarily clinical (e.g., treating iron deficiency anemia) and should be individualized, while prevention-oriented applications remain investigational and require prospective validation [74,79,81]. Accordingly, iron metabolism should be presented as a translational avenue for refining personalized prevention hypotheses, potentially informing lifestyle counseling and risk-contextual decision-making, rather than as an evidence-based preventive target.

## 6. Future Perspectives

Growing evidence indicates that iron metabolism plays a multifaceted role in CRC, extending beyond systemic iron homeostasis to influence the TME, immune regulation, oxidative stress, and ferroptosis susceptibility. However, despite considerable advances in understanding these mechanisms, their translation into routine clinical practice remains limited.

Future research should prioritize prospective multicenter studies integrating systemic, tissue, and spatial biomarkers of iron metabolism with molecular tumor characteristics, including microsatellite status, consensus molecular subtypes, and tumor sidedness. The development of standardized analytical methods and clinically validated cutoff values for iron-related biomarkers, particularly ferritin, hepcidin, sTfR and tissue iron markers, will be essential to improve their diagnostic and prognostic utility.

Advances in spatial transcriptomics, single-cell sequencing, and artificial intelligence-assisted pathology offer new opportunities to characterize iron metabolism within the colorectal TME at unprecedented resolution. These approaches may facilitate the identification of patient subgroups most likely to benefit from iron-targeted therapeutic strategies.

Finally, future clinical trials should evaluate not only the efficacy of iron supplementation for correcting iron deficiency but also its long-term effects on the TME, immune responses, microbiota composition, and oncologic outcomes. A more comprehensive understanding of iron biology may ultimately support the development of personalized therapeutic approaches integrating iron metabolism into precision oncology for CRC.

## 7. Conclusions

This review highlights that iron dysregulation is a consistent feature across the colorectal neoplasia continuum and has meaningful clinical implications. Alterations in iron metabolism are detectable from the stage of adenomas and become more pronounced with tumor progression, supporting a role for iron in both early tumorigenesis and advanced disease biology. A characteristic finding is the coexistence of systemic iron deficiency with intratumoral iron accumulation, driven largely by inflammation-mediated dysregulation of the hepcidin–ferroportin axis.

A broad range of iron-related biomarkers has been investigated. Systemic markers such as serum iron, ferritin, TSAT, and sTfR reflect iron availability but are variably influenced by inflammation. TSAT and sTfR appear more reliable for identifying functional iron deficiency, whereas ferritin requires cautious interpretation. At the tissue level, overexpression of TfR1, impaired ferroportin function, STEAP4 upregulation, and intratumoral iron deposition collectively indicate increased iron retention and metabolic adaptation within tumor cells. In parallel, oxidative stress (e.g., PCO) and inflammatory markers (CRP, IL-6) further characterize the iron–inflammation axis in CRC.

Clinically, iron status is associated with tumor aggressiveness and outcomes. Iron deficiency correlates with advanced stage, poorer differentiation, reduced response to neoadjuvant therapy, and delayed postoperative recovery, while markers of iron excess are also linked to worse survival, suggesting a U-shaped prognostic relationship. In preneoplastic disease, TSAT has shown potential associations with adenoma risk, although its screening utility remains limited.

From a management perspective, iron supplementation remains essential in confirmed iron deficiency anemia, particularly perioperatively, but should be individualized. In FID or non-anemic patients, indiscriminate supplementation may be ineffective or potentially harmful due to effects on oxidative stress and the TME.

Overall, iron-related biomarkers offer complementary diagnostic and prognostic value, especially when integrated with clinical, endoscopic, and histological data. However, their routine implementation is limited by assay variability, lack of standardized thresholds, and insufficient prospective validation. Future research should focus on multimarker approaches and clinically applicable algorithms to better define the role of iron metabolism in risk stratification and personalized management of CRC.

## Data Availability

No new data were created or analyzed in this study. Data sharing is not applicable to this article.

## Abbreviations

The following abbreviations are used in this manuscript:

AID	absolute iron deficiency
Arg1	arginase 1
CEA	carcinoembryonic antigen
CIMP	CpG island methylator phenotype
CIN	chromosomal instability
CRC	colorectal cancer
CRP	C-reactive protein
DMT1	divalent metal transporter 1
DNA	deoxyribonucleic acid
Fe^2+^	ferrous iron
Fe^3+^	ferric iron
FID	functional iron deficiency
FIT	fecal immunochemical test
IL-6	interleukin-6
MSI	microsatellite instability
NHANES	National Health and Nutrition Examination Survey
NTBI	non-transferrin-bound iron
PCO	protein carbonyls
ROS	reactive oxygen species
sTfR	soluble transferrin receptor
STEAP4	six-transmembrane epithelial antigen of prostate 4
TAMs	tumor-associated macrophages
TfR1	transferrin receptor 1
TME	tumor microenvironment
TSAT	transferrin saturation
Ym1	chitinase-like protein Ym1

## References

[1] Huang L., Li W., Lu Y., Ju Q., Ouyang M. (2023). Iron Metabolism in Colorectal Cancer. Front. Oncol..

[2] Yan H., Talty R., Aladelokun O., Bosenberg M., Johnson C.H. (2023). Ferroptosis in Colorectal Cancer: A Future Target?. Br. J. Cancer.

[3] Ahmadi N., Vidanapathirana G., Gopalan V. (2025). Crossroads of Iron Metabolism and Inflammation in Colorectal Carcinogenesis: Molecular Mechanisms and Therapeutic Perspectives. Genes.

[4] Folkert I.W., Molina Arocho W.A., To T.K.J., Devalaraja S., Molina I.S., Shoush J., Mohei H., Zhai L., Akhtar M.N., Kochat V. (2024). An Iron-Rich Subset of Macrophages Promotes Tumor Growth through a Bach1-Ednrb Axis. J. Exp. Med..

[5] Aceto G.M., Catalano T., Curia M.C. (2020). Molecular Aspects of Colorectal Adenomas: The Interplay among Microenvironment, Oxidative Stress, and Predisposition. BioMed Res. Int..

[6] Bu X., Wang L. (2024). Iron Metabolism and the Tumor Microenvironment: A New Perspective on Cancer Intervention and Therapy. Int. J. Mol. Med..

[7] Luo Y., Zheng P., Luo H., Chen X., Zhang K., Wang W., Song B., Liu C., Rao L., Yang H. (2025). Impact of Iron Deficiency on Therapeutic Outcomes in Colorectal Cancer Patients: A Single-Center Cohort Study. J. Transl. Med..

[8] Mleczko-Sanecka K., Silvestri L. (2021). Cell-type-specific Insights into Iron Regulatory Processes. Am. J. Hematol..

[9] Tomoiagă A.-V., Suciu Șoimița M., Gerdanovics C.-A., Gerdanovics A., Milaciu M.-V., Perne M.-G., Alexescu T.-G., Ciumărnean L., Cozma A., Negrean V. (2026). Iron Metabolism in the Colorectal Tumor Microenvironment: From Preneoplastic Lesions to Cancer Progression. Int. J. Mol. Sci..

[10] Di Grazia A., Di Fusco D., Franzè E., Colella M., Strimpakos G., Salvatori S., Formica V., Laudisi F., Maresca C., Colantoni A. (2022). Hepcidin Upregulation in Colorectal Cancer Associates with Accumulation of Regulatory Macrophages and Epithelial–Mesenchymal Transition and Correlates with Progression of the Disease. Cancers.

[11] Liu X., Zhang X., Fan Y., Tan K. (2025). Hepcidin: A Multifaceted Hormone in Iron Homeostasis and Tumor Biology. Vitam. Horm..

[12] Frascatani R., Colella M., Monteleone G. (2024). Hepcidin Is a Valuable Therapeutic Target for Colorectal Cancer. Cancers.

[13] Basak T., Kanwar R.K. (2022). Iron Imbalance in Cancer: Intersection of Deficiency and Overload. Cancer Med..

[14] Shen L., Zhou Y., He H., Chen W., Lenahan C., Li X., Deng Y., Shao A., Huang J. (2021). Crosstalk between Macrophages, T Cells, and Iron Metabolism in Tumor Microenvironment. Oxid. Med. Cell. Longev..

[15] Chen D., Jiang X., Duan T., Tian Y., Zhang J., Wang X., Tan J. (2026). NCOA4-Mediated Ferritinophagy: Emerging Role and Novel Therapeutic Target in Precision Oncology. Autophagy.

[16] Hoelzgen F., Nguyen T.T.P., Klukin E., Boumaiza M., Srivastava A.K., Kim E.Y., Zalk R., Shahar A., Cohen-Schwartz S., Meyron-Holtz E.G. (2024). Structural Basis for the Intracellular Regulation of Ferritin Degradation. Nat. Commun..

[17] Liu L., Liu Y., Zhou X., He H., Chen N., Qin Y., Sun X., Bian Z., Zhang Q., Mao L. (2025). Sodium Butyrate Induces Ferroptosis in Colorectal Cancer Cells by Promoting NCOA4-FTH1-Mediated Ferritinophagy. Int. Immunopharmacol..

[18] Chabane T., Bouscary D., Grignano E. (2026). NCOA4 and Ferritinophagy in Hematological Malignancies: A Double-Edged Regulator of Iron Metabolism and Cell Fate. Front. Oncol..

[19] Jin W., Xia J., Zhang C., Shi T., Shen Y. (2026). Unlocking Ferroptosis to Overcome Cancer Stem Cells-Mediated Treatment Failure and Immune Evasion. Front. Immunol..

[20] Battaglia A.M., Sacco A., Vecchio E., Scicchitano S., Petriaggi L., Giorgio E., Bulotta S., Levi S., Faniello C.M., Biamonte F. (2023). Iron Affects the Sphere-Forming Ability of Ovarian Cancer Cells in Non-Adherent Culture Conditions. Front. Cell Dev. Biol..

[21] Badr O.I., Mustafa F.A., Radwan B.A., Abdulfatah A.M., Ragab A.N., Sleem H.M. (2025). Ferritinophagy as a Double-Edged Sword in Cancer: Novel Insights into Therapeutic Targeting of Iron-Driven Ferroptosis. Cell Biochem. Biophys..

[22] Schöttker B., Gào X., Jansen E.H., Brenner H. (2021). Associations of Human Colorectal Adenoma with Serum Biomarkers of Body Iron Stores, Inflammation and Antioxidant Protein Thiols. Antioxidants.

[23] Cao C., Tang F., Zhu Z., Wang F., Li J. (2026). Serum Ferritin as a Potential Biomarker for Distinguishing Advanced-Stage Colorectal Cancer: A Retrospective Cohort Study. PeerJ.

[24] Urback A.L., Martens K., McMurry H.S., Sharma A., Citti C., DeLoughery T.G., Shatzel J.J. (2024). Serum Ferritin and Risk of Colonic Neoplasia: Implications for the Workup and Treatment of Iron Deficiency. Eur. J. Haematol..

[25] Ahmed I., Bachiashvili K., Baird J., Bakhshi S., Chheng E., Cool R., Dickerson T., Dinner S., Lurie R.H., Fallon M. (2025). NCCN Guidelines Version 3.2026 Hematopoietic Growth Factors.

[26] Formica V., Di Grazia A., Bonomo M.V., Frascatani R., Mancone R., Monteleone G. (2024). Circulating Hepcidin Levels Are an Independent Predictor of Survival in Microsatellite Stable Metastatic Colorectal Cancer Patient Candidates for Standard First-Line Treatment. Cancers.

[27] Shao Y., Chen C., Zhang X., Liu N., Fan X., Tan X.Y., Deng B., Gao R., Shi S., Zhao H. (2026). Integrative Genomic Profiling of Iron Homeostasis Predicts Clinical Outcomes and Identifies HAMP/Hepcidin as a Therapeutic Target in Colorectal Cancer. Cell Biosci..

[28] Brookes M.J., Hughes S., Turner F.E., Reynolds G., Sharma N., Ismail T., Berx G., McKie A.T., Hotchin N., Anderson G.J. (2006). Modulation of Iron Transport Proteins in Human Colorectal Carcinogenesis. Gut.

[29] Yin K., Villareal L., Wu X., Arcos M., Lee J., Martin D.R., In J.G., Leslie K., Zhang D.D., Xue X. (2025). The STEAP4 Target NQO1 Mediates Colon Tumorigenesis. J. Cell Sci..

[30] Ploug M., Kroijer R., Qvist N., Lindahl C.H., Knudsen T. (2021). Iron Deficiency in Colorectal Cancer Patients: A Cohort Study on Prevalence and Associations. Colorectal Dis..

[31] Sulaiman S.H., Ali H.S., Omer R.A., Barzani H.A.H., Salih M.I., Qader A.F. (2025). Biochemical Insights into Oxidative Stress in Colon Cancer Patients. Cell Biochem. Biophys..

[32] Ness R.M., Llor X., Chair V., Baidoo L., Jude S., Bishu S., Cooper G., Early D.S., Friedman M., Fudman D. (2025). NCCN Guidelines Version 2.2025 Colorectal Cancer Screening.

[33] Cao H., Wang C., Chai R., Dong Q., Tu S. (2017). Iron Intake, Serum Iron Indices and Risk of Colorectal Adenomas: A Meta-Analysis of Observational Studies. Eur. J. Cancer Care.

[34] Wilhelmsen M., Christensen I.J., Rasmussen L., Jørgensen L.N., Madsen M.R., Vilandt J., Hillig T., Klaerke M., Nielsen K.T., Laurberg S. (2017). Detection of Colorectal Neoplasia: Combination of Eight Blood-Based, Cancer-Associated Protein Biomarkers. Int. J. Cancer.

[35] Sawayama H., Miyamoto Y., Mima K., Kato R., Ogawa K., Hiyoshi Y., Shimokawa M., Akiyama T., Kiyozumi Y., Iwagami S. (2021). Preoperative Iron Status Is a Prognosis Factor for Stage II and III Colorectal Cancer. Int. J. Clin. Oncol..

[36] Malesza I.J., Bartkowiak-Wieczorek J., Winkler-Galicki J., Nowicka A., Dzięciołowska D., Błaszczyk M., Gajniak P., Słowińska K., Niepolski L., Walkowiak J. (2022). The Dark Side of Iron: The Relationship between Iron, Inflammation and Gut Microbiota in Selected Diseases Associated with Iron Deficiency Anaemia—A Narrative Review. Nutrients.

[37] Pfeiffer C.M., Looker A.C. (2017). Laboratory Methodologies for Indicators of Iron Status: Strengths, Limitations, and Analytical Challenges. Am. J. Clin. Nutr..

[38] Gwenzi T., Schrotz-King P., Anker S.C., Schöttker B., Hoffmeister M., Brenner H. (2024). Prognostic Value of Post-Operative Iron Biomarkers in Colorectal Cancer: Population-Based Patient Cohort. Br. J. Cancer.

[39] Szymulewska-Konopko K., Reszeć-Giełażyn J., Małeczek M. (2025). Ferritin as an Effective Prognostic Factor and Potential Cancer Biomarker. Curr. Issues Mol. Biol..

[40] Cui C., Cheng X., Yan L., Ding H., Guan X., Zhang W., Tian X., Hao C. (2019). Downregulation of TfR1 Promotes Progression of Colorectal Cancer via the JAK/STAT Pathway. Cancer Manag. Res..

[41] Yamane T., Kanamori Y., Sawayama H., Yano H., Nita A., Ohta Y., Hinokuma H., Maeda A., Iwai A., Matsumoto T. (2022). Iron Accelerates Fusobacterium Nucleatum–Induced CCL8 Expression in Macrophages and Is Associated with Colorectal Cancer Progression. JCI Insight.

[42] Jiang J., Wang K., Chen Y., Chen H., Nice E.C., Huang C. (2017). Redox Regulation in Tumor Cell Epithelial–Mesenchymal Transition: Molecular Basis and Therapeutic Strategy. Signal Transduct. Target. Ther..

[43] Huang Q., Jing Y., Xiong L., Li L., Feng J., Cheng J. (2025). The Interplay between Driver Mutation and Oxidative Stress in Colorectal Cancer: From Pathogenesis to Therapeutics. J. Transl. Med..

[44] Ganz T. (2025). Systemic Iron Metabolism.

[45] Wilson M.J., Dekker J.W.T., Harlaar J.J., Jeekel J., Schipperus M., Zwaginga J.J. (2017). The Role of Preoperative Iron Deficiency in Colorectal Cancer Patients: Prevalence and Treatment. Int. J. Colorectal Dis..

[46] Bhurosy T., Jishan A., Boland P.M., Lee Y.-H., Heckman C.J. (2022). Underdiagnosis of Iron Deficiency Anemia among Patients with Colorectal Cancer: An Examination of Electronic Medical Records. BMC Cancer.

[47] Chardalias L., Papaconstantinou I., Gklavas A., Politou M., Theodosopoulos T. (2023). Iron Deficiency Anemia in Colorectal Cancer Patients: Is Preoperative Intravenous Iron Infusion Indicated? A Narrative Review of the Literature. Cancer Diagn. Progn..

[48] Făgărășan V., Andraș D., Amarinei G., Seicean R.I., Bințințan V.V., Dindelegan G.C., Căinap C.I. (2022). Absolute and Functional Iron Deficiency in Colon Cancer: A Cohort Study. Medicina.

[49] Köseoğlu F.D., Tuğral A., Akyol M. (2026). A Prospective Study of Intravenous Iron Effectiveness on Quality of Life and Functional Outcomes in Patients with Cancer. Sci. Rep..

[50] Estêvão D., da Cruz-Ribeiro M., Cardoso A.P., Costa Â.M., Oliveira M.J., Duarte T.L., da Cruz T.B. (2023). Iron Metabolism in Colorectal Cancer: A Balancing Act. Cell. Oncol..

[51] Qi J., Liang Y., Yu D., Li W., Long F., Yuan M., Lou Z., Liu C., Wang G., Wu B. (2026). Iron Deficiency Aggravates Hepatic Inflammation in Suckling Piglets via Endoplasmic Reticulum Stress-Driven NF-ΚB Pathway Activation. J. Anim. Sci. Biotechnol..

[52] Lund E.K., Wharf S.G., Fairweather-Tait S.J., Johnson I.T. (1999). Oral Ferrous Sulfate Supplements Increase the Free Radical–Generating Capacity of Feces from Healthy Volunteers. Am. J. Clin. Nutr..

[53] Chen S., Ma J., Tang J., Yang Y., Zhou S., Feng P. (2025). Research Progress of Macrophage Ferroptosis in Inflammatory Bowel Disease and Inflammation-Cancer Transformation. Front. Immunol..

[54] Pantopoulos K. (2024). Oral Iron Supplementation: New Formulations, Old Questions. Haematologica.

[55] Babayev M., Klaunig J., Silveyra P., Henschel B., Gletsu-Miller N. (2023). Impact on Oxidative Stress of Oral, High-Dose, Iron Supplementation for Management of Iron Deficiency after Bariatric Surgery, a Preliminary Study. J. Trace Elem. Med. Biol..

[56] Baser M.N. (2025). Impact of Oral Iron Therapy on Oxidative Stress and DNA Damage in Women of Reproductive Age. J. Clin. Pract. Res..

[57] Vaucher P., Druais P.-L., Waldvogel S., Favrat B. (2012). Effect of Iron Supplementation on Fatigue in Nonanemic Menstruating Women with Low Ferritin: A Randomized Controlled Trial. Can. Med. Assoc. J..

[58] Verdon F., Burnand B., Stubi C.-L.F., Bonard C., Graff M., Michaud A., Bischoff T., de Vevey M., Studer J.-P., Herzig L. (2003). Iron Supplementation for Unexplained Fatigue in Non-Anaemic Women: Double Blind Randomised Placebo Controlled Trial. BMJ.

[59] Nemeth E., Ganz T. (2006). Regulation of Iron Metabolism by Hepcidin. Annu. Rev. Nutr..

[60] Angoro B., Motshakeri M., Hemmaway C., Svirskis D., Sharma M. (2022). Non-Transferrin Bound Iron. Clin. Chim. Acta.

[61] Agoro R., Taleb M., Quesniaux V.F.J., Mura C. (2018). Cell Iron Status Influences Macrophage Polarization. PLoS ONE.

[62] Sacco A., Battaglia A.M., Botta C., Aversa I., Mancuso S., Costanzo F., Biamonte F. (2021). Iron Metabolism in the Tumor Microenvironment—Implications for Anti-Cancer Immune Response. Cells.

[63] Cuisiniere T., Hajjar R., Oliero M., Calvé A., Fragoso G., Rendos H.V., Gerkins C., Taleb N., Gagnon-Konamna M., Dagbert F. (2025). Initial Gut Microbiota Composition Is a Determining Factor in the Promotion of Colorectal Cancer by Oral Iron Supplementation: Evidence from a Murine Model. Microbiome.

[64] Swain I.X., Kresak A.M. (2024). Iron Supplementation Increases Tumor Burden and Alters Protein Expression in a Mouse Model of Human Intestinal Cancer. Nutrients.

[65] Zhang Q., Wu W., Guo F., Li J., Jin Y., Cai G., Yang Y. (2024). Characteristics of Gut Microbiota and Fecal Metabolites in Patients with Colorectal Cancer-Associated Iron Deficiency Anemia. Microorganisms.

[66] Cozac-Szoke A.-R., Cotoi O.S., Mauer U., Steinestel K., Arndt A. (2026). Integrated Molecular Profiling of Colorectal Cancer by Tumor Location: Evidence from a Real-World Cohort with Primary and Metastatic Samples. Cancers.

[67] Huang W., Li W., Xu N., Li H., Zhang Z., Zhang X., He T., Yao J., Xu M., He Q. (2023). Differences in DNA Damage Repair Gene Mutations between Left- and Right-sided Colorectal Cancer. Cancer Med..

[68] Oliveira D.M., Laudanna C., Migliozzi S., Zoppoli P., Santamaria G., Grillone K., Elia L., Mignogna C., Biamonte F., Sacco R. (2018). Identification of Different Mutational Profiles in Cancers Arising in Specific Colon Segments by next Generation Sequencing. Oncotarget.

[69] Petrie A., Carson D.A., Cribb B. (2026). Comparison of Histopathologic Features of Right and Left Sided Colon Cancer. ANZ J. Surg..

[70] Vidanapathirana G., Islam M.S., Gamage S., Lam A.K., Gopalan V. (2025). The Role of Iron Chelation Therapy in Colorectal Cancer: A Systematic Review on Its Mechanisms and Therapeutic Potential. Cancer Med..

[71] Xue X., Ramakrishnan S.K., Weisz K., Triner D., Xie L., Attili D., Pant A., Győrffy B., Zhan M., Carter-Su C. (2016). Iron Uptake via DMT1 Integrates Cell Cycle with JAK-STAT3 Signaling to Promote Colorectal Tumorigenesis. Cell Metab..

[72] Aljehani M.A., Morgan J.W., Guthrie L.A., Jabo B., Ramadan M., Bahjri K., Lum S.S., Selleck M., Reeves M.E., Garberoglio C. (2018). Association of Primary Tumor Site With Mortality in Patients Receiving Bevacizumab and Cetuximab for Metastatic Colorectal Cancer. JAMA Surg..

[73] Lee M.S., Menter D.G., Kopetz S. (2017). Right Versus Left Colon Cancer Biology: Integrating the Consensus Molecular Subtypes. J. Natl. Compr. Cancer Netw..

[74] Tsokkou S., Konstantinidis I., Papakonstantinou M., Chatzikomnitsa P., Liampou E., Toutziari E., Giakoustidis D., Bangeas P., Papadopoulos V., Giakoustidis A. (2025). Sex Differences in Colorectal Cancer: Epidemiology, Risk Factors, and Clinical Outcomes. J. Clin. Med..

[75] Boka H.J., Engel R.M., Georges C., McMurrick P.J., Abud H.E. (2025). Does Side Matter? Deciphering Mechanisms That Underpin Side-Dependent Pathogenesis and Therapy Response in Colorectal Cancer. Mol. Cancer.

[76] Zhou M., Shao Y., Chen W., Guan B., Xie B., Liu Y., Gu Q., Zhou M., Peng D., Li F. (2024). Association between Serum Iron Status and the Risk of Colorectal Cancer in US Adults: A Cross-Sectional Analysis of NHANES 2001–2020. BMC Gastroenterol..

[77] Jung Y.S. (2023). Summary and Comparison of Recently Updated Post-Polypectomy Surveillance Guidelines. Intest. Res..

[78] Sadowski D.C., Kolber M.R., Gomes A., Hickle L., Hilsden R., McLean D.R., Mok D., Moysey B., Nemecek N., Ryan J.D. (2024). Post-Polypectomy Surveillance: Follow-up Recommendations from the Alberta Colorectal Cancer Screening Program. J. Can. Assoc. Gastroenterol..

[79] Yousefi M.H., Masoudi A., Saberi Rounkian M., Mansouri M., Hojat B., Kaveh Samani M., Veisi R., Honarvar Bakeshloo P., Nosratipour R., Afkhami H. (2025). An Overview of the Current Evidences on the Role of Iron in Colorectal Cancer: A Review. Front. Oncol..

[80] Rizzolo-Brime L., Farran-Codina A., Bou R., Luján-Barroso L., Quirós J.R., Amiano P., Sánchez M.J., Rodríguez-Barranco M., Guevara M., Moreno-Iribas C. (2024). Nitrosyl-Heme and Heme Iron Intake from Processed Meats in Subjects from the EPIC-Spain Cohort. Nutrients.

[81] Zeidan R.S., Yoon H.-S., Yang J.J., Sobh A., Braithwaite D., Mankowski R., Leeuwenburgh C., Anton S. (2024). Iron and Cancer: Overview of the Evidence from Population-Based Studies. Front. Oncol..

